# A New CYP2E1 Inhibitor, 12-Imidazolyl-1-dodecanol, Represents a Potential Treatment for Hepatocellular Carcinoma

**DOI:** 10.1155/2021/8854432

**Published:** 2021-02-02

**Authors:** Torsten Diesinger, Alfred Lautwein, Sebastian Bergler, Dominik Buckert, Christian Renz, Radovan Dvorsky, Vyacheslav Buko, Siarhei Kirko, Edith Schneider, Florian Kuchenbauer, Mukesh Kumar, Cagatay Günes, Felicitas Genze, Berthold Büchele, Thomas Simmet, Martin Haslbeck, Kai Masur, Thomas Barth, Dieter Müller-Enoch, Thomas Wirth, Thomas Haehner

**Affiliations:** ^1^Chair of Biochemistry and Molecular Medicine, Witten/Herdecke University, Faculty of Health/School of Medicine, Alfred-Herrhausen-Straße 50, 58448 Witten, Germany; ^2^Institute of Physiological Chemistry, University of Ulm, Albert-Einstein-Allee 11, 89081 Ulm, Germany; ^3^Department of Internal Medicine, Neu-Ulm Hospital, Krankenhausstraße 11, 89231 Neu-Ulm, Germany; ^4^Department of Internal Medicine II, University Hospital Ulm, Albert-Einstein-Allee 23, 89081 Ulm, Germany; ^5^Institute of Biochemistry and Molecular Biology II, Medical Faculty of the Heinrich Heine University Düsseldorf, Moorenstraße 5, 40225 Düsseldorf, Germany; ^6^Max Planck Institute of Molecular Physiology, Otto-Hahn-Straße 11, 44227 Dortmund, Germany; ^7^Division of Biochemical Pharmacology, Institute of Biochemistry of Biologically Active Compounds, National Academy of Sciences, Bulvar Leninskogo Komsomola, Dom 50, Grodno 230030, Belarus; ^8^Department of Biotechnology, University of Medical Sciences, Ulica Jana Kilinskiego 1, 15-089 Białystok, Poland; ^9^Department of Internal Medicine III, University Hospital Ulm, Albert-Einstein-Allee 23, 89081 Ulm, Germany; ^10^University of British Columbia, Terry Fox Laboratory, 675 West 10th Avenue, Vancouver, BC V5Z 1L3, Canada; ^11^Department of Urology, University Hospital Ulm, Albert-Einstein-Allee 23, 89081 Ulm, Germany; ^12^Institute of Pharmacology of Natural Products and Clinical Pharmacology, University Ulm, Helmholtzstraße 20, 89081 Ulm, Germany; ^13^Chair of Biotechnology, TUM Department of Chemistry, Technical University of Munich, Lichtenbergstraße 4, 85748 Garching, Munich, Germany; ^14^Leibniz Institute for Plasma Science and Technology, Felix-Hausdorff-Straße 2, 17489 Greifswald, Germany; ^15^Institute of Pathology, Ulm University, Albert-Einstein-Allee 23, 89081 Ulm, Germany

## Abstract

Cytochrome P450 2E1 (CYP2E1) is a key target protein in the development of alcoholic and nonalcoholic fatty liver disease (FLD). The pathophysiological correlate is the massive production of reactive oxygen species. The role of CYP2E1 in the development of hepatocellular carcinoma (HCC), the final complication of FLD, remains controversial. Specifically, CYP2E1 has not yet been defined as a molecular target for HCC therapy. In addition, a CYP2E1-specific drug has not been developed. We have already shown that our newly developed CYP2E1 inhibitor 12-imidazolyl-1-dodecanol (I-ol) was therapeutically effective against alcoholic and nonalcoholic steatohepatitis. In this study, we investigated the effect of I-ol on HCC tumorigenesis and whether I-ol could serve as a possible treatment option for terminal-stage FLD. I-ol exerted a very highly significant antitumour effect against hepatocellular HepG2 cells. Cell viability was reduced in a dose-dependent manner, with only the highest doses causing a cytotoxic effect associated with caspase 3/7 activation. Comparable results were obtained for the model colorectal adenocarcinoma cell line, DLD-1, whose tumorigenesis is also associated with CYP2E1. Transcriptome analyses showed a clear effect of I-ol on apoptosis and cell-cycle regulation, with the increased expression of p27Kip1 being particularly noticeable. These observations were confirmed at the protein level for HepG2 and DLD-1 cells grafted on a chorioallantoic membrane. Cell-cycle analysis showed a complete loss of proliferating cells with a simultaneous increase in S-phase arrest beginning at a threshold dose of 30 *μ*M. I-ol also reduced xenograft tumour growth in nude mice. This antitumour effect was not associated with tumour cachexia. I-ol was not toxic to healthy tissues or organs. This study demonstrates for the first time the therapeutic effect of the specific CYP2E1 inhibitor I-ol on the tumorigenesis of HCC. Our findings imply that I-ol can potentially be applied therapeutically on patients at the final stage of FLD.

## 1. Introduction

Hepatocellular carcinoma (HCC) is the fifth most common malignant tumour worldwide and is responsible for every fourth death of tumour patients [1]. The number of new cases is rising rapidly worldwide, especially in western industrialized countries. Between 70 and 90% of HCC cases are caused by chronic liver diseases such as alcoholic steatohepatitis (ASH) and nonalcoholic steatohepatitis (NASH).

At present, there are no drugs that, alone or in combination with other chemotherapeutic agents, can lead to an outstanding overall survival rate. Liver transplantation is often the only curative method but is only recommended within a narrowly defined diagnosis window.

However, a general trend exists in that new antitumour drugs are increasingly replacing traditional chemotherapeutic agents, such as cisplatin, doxorubicin, 5-fluorouracil, and its prodrug capecitabine.

Sorafenib as a targeted chemotherapeutic for treating HCC prolongs survival time by an average of only three months [2]. Lenvatinib is noninferior to sorafenib [3] and was approved as a first-line treatment by the US Food and Drug Administration and European Commission. Regorafenib [4] and cabozantinib [5] were approved as alternatives and second-line treatments after sorafenib. Other promising drug candidates, such as the orally available selective MET (mesenchymal-epithelial transition factor receptor) inhibitor tivantinib, have failed to pass the next milestone in clinical development [6, 7]. Due to the low level of innovation in the development of new drugs, other therapeutic approaches are being pursued in parallel, which rely on classic chemotherapeutic agents. Capecitabine is a representative of the concept of metronomic chemotherapy, according to which the therapeutic agent is administered continuously but in a reduced dose [8, 9].

Epidemiological data show that HCC development is closely linked to chronic liver damage, in particular cirrhosis. Hepatocyte proliferation increases during the stage of chronic hepatitis, while the subsequent stage of liver cirrhosis is associated with reduced hepatocyte proliferation [10].

However, hepatocarcinogenesis is promoted in remodelled cirrhotic livers, as follows: (1) alterations in genes encoding DNA-damage and apoptosis checkpoints (such as p14, Rb, p16, p21, p27, p53, gankyrin, IGF2R, ARF, and MDM2) in genetically modified hepatocytes can potentially provide them with a selective growth advantage [11]. (2) The simultaneous activation of stellate cells and the infiltration of inflammatory cells leads to increased production of extracellular matrix proteins, cytokines, growth factors, and reactive oxygen species (ROS) [12], which alter the proliferation of hepatocytes and promote tumour formation [13]. (3) The deterioration of liver function leads to increased levels of toxic metabolites in the blood, which can lead to a growth-promoting macroenvironment through the activation of feedback mechanisms.

CYP2E1 does not belong to those isoenzymes of drug-metabolizing cytochromes that play a role in the hepatic metabolism of antitumour drugs. Dacarbazine (DTIC), which is used for the treatment of malignant melanoma and Hodgkin's lymphoma, is the only chemotherapeutic agent known to date that is metabolically activated by CYP2E1, but only at elevated concentrations [14]. In contrast, the role of CYP2E1 in hepatogenesis is not yet fully understood. Besides the enzymatic catalysis of a large number of low-molecular procarcinogenic substances into active carcinogens by CYP2E1 [15, 16], its expression shows a remarkable distribution pattern. CYP2E1 expression is the strongest in peritumourous cirrhotic tissue, compared to the noncirrhotic and malignant areas [17, 18]. Because strong CYP2E1 expression drives tumour cells of the human liver into apoptosis [19], it seems likely that the increased expression of the enzyme in the cirrhotic liver and the associated production of ROS and carcinogens leads to an increased rate of malignant neoplasia.

Despite the recognized importance of CYP2E1 in the pathogenesis of the alcoholic and nonalcoholic fatty liver disease (FLD), where HCC is the final stage, CYP2E1 has not yet been evaluated as a pharmacological target. We developed a highly competitive CYP2E1 inhibitor 12-imidazolyl-1-dodecanol (I-ol) (Table S1) and have already demonstrated proof-of-concept that it can successfully be used to treat ASH (Figure S1) and NASH (Figure S2). The findings of this study indicate that I-ol is effective against hepatocarcinogenesis, and our findings imply that I-ol could be potentially used to treat FLD.

## 2. Materials and Methods

### 2.1. Cancer Cell Grafting

A silicone ring was placed in the centre of the chorioallantoic membrane (CAM) of fertilized chicken eggs (Lohmann LSL-Classic, Lohmann Tierzucht, Germany) on the 8th day of incubation. Immediately afterwards, HepG2 cells (10^6^; ATCC®: HB-8065™) were dispersed in 20 *μ*L of a medium-Matrigel (BD Biosciences, USA) solution (1 : 1 volume ratio) and were placed within the ring.

### 2.2. CAM Model

Therapy was started by administering 10 *μ*L I-ol to CAMs at concentrations of 1, 3, 10, and 30 *μ*M. An aqueous 10 *μ*M solution of the corresponding cage-like carrier substance, hydroxy-propyl-beta-cyclodextrin (HP*β*CD, referred to here as CD) that captures I-ol very effectively, formed the control group (Figure S3).

The solutions were administered once daily for three days (9th–11th day of incubation). On day five (12th day of incubation), each CAM was fixed in 4% formaldehyde for further histological evaluation. All positively stained cells were counted at 400×, under high-power field magnification, using a light microscope (Zeiss, Germany) [20].

Experiments performed with CAMs and mice are represented in Figure S4.

### 2.3. Xenograft Animal Model

The care, daily handling, and experiments carried out with the animals conformed to the institutional guidelines, national and international laws, and Directive 2010/63/EU of the European Parliament and the Council of 22.10.2010 on the protection of animals used for scientific purposes. The animal study was approved by the animal-welfare officers, an internal animal-welfare commission, and the concerned authorities after following a standardized procedure to verify the scientific, animal welfare, ethical, and statistical statements.

A proof-of-concept trial with our mouse model was conducted by performing two separate animal studies, for technical reasons. We used 34 male, NMRI Nu/Nu athymic nude mice (Janvier, Le Genest, France), aged 6–8 weeks, with an initial body weight of 25–35 g. Each mouse was housed in a separate cage with a controlled experimental environment (20–25°C, 40–50% humidity, and a 12 h light/dark cycle) with free access to food during the whole study period. The mice were adapted to the housing conditions over seven days before the animal studies were started.

On day 1, 1.5 × 10^6^ HepG2 cells were dissolved in 50 *μ*L Dulbecco's Modified Eagle's Medium and mixed with an equal volume of Matrigel. This step was necessary so that the HepG2 cells could be transplanted into a foreign tissue. The total volume of the cell-Matrigel mixture (100 *μ*L) was injected subcutaneously behind both shoulder blades immediately thereafter.

The mice were given four days to adapt to the transplanted cell mixture and were then randomly divided into three experimental groups consisting of one comparison group and two therapy groups (Figure S5). The comparison group received the carrier substance CD at a dose of 3 mg/kg body weight (bw), and the two therapy groups received the CYP2E1 inhibitor I-ol at a dose of 1 or 3 mg/kg bw, via intravenous application into the tail vein, once daily for 15 days.

Tumour transplantation and daily injections were performed under short anaesthesia with forene (isoflurane, Abbott, USA). Mouse weights, tumour sizes, and the summed volume of both tumours in each mouse were evaluated at the same time. The measurements began with the start of therapy on day 5 and then during therapy on days 8, 12, 15, and 19. After 20 days, the mice were sacrificed under fasting conditions via aorta dissection and exsanguination, after receiving an overdose of isoflurane. The tumours, as well as the heart, liver, spleen, and kidneys, were removed during the autopsies. This was followed by the determination of the final tumour weights by summing both values for each mouse.

All extracted tissues were fixed in 4% formaldehyde for further histological and immunohistochemical processing.

### 2.4. Viability, Cytotoxicity, and Caspase-Activity Assays in HepG2 and DLD-1 Cells

Kits from Promega (USA) were used to determine the time- and dose-dependent relationships for I-ol and CD in terms of cell viability, cytotoxicity, and apoptosis induction via caspase 3/7 activation. The CellTiter Glo® Kit was used to determine the viabilities of HepG2 and DLD-1 cells (ATCC® CCL-221™). The CellTiter Blue® Kit was used to determine the effect of cyclodextrin and to initially determine the effect of small I-ol concentrations on the viability of HepG2 cells. The Cytotox Glo® Kit was used to exclude cytotoxic effects on HepG2 cells. The Caspase Glo 3/7® Kit was used to measure caspase 3/7 activity. The reagents were used according to the manufacturer's specifications in each respective manual.

The experimental design of the experiments was as follows. The cells were initially seeded in 96-well tissue-culture plates at a density of 5 × 10^3^ HepG2 cells/well or 3 × 10^3^ DLD-1 cells/well, based on their different growth rates. After a 24-hour acclimation phase, the cells were incubated with I-ol or CD at final concentrations of 0.1, 1, 3, 10, 30, 100, or 200 *μ*M, and luminescence measurements were taken after 1, 2, 4, 8, 12, 16, 24, 48, 72, or 96 h in a GloMax-96 Microplate Luminometer (Promega, USA), following the manufacturer's user manual. During the experiments, each indicated reagent was added, and the plates were gently shaken and incubated at an average room temperature of 21°C for the recommended time.

### 2.5. mRNA Expression of Genes Related to Apoptosis and Cell-Cycle Progression in HepG2 Cells

HepG2 cells (2 × 10^6^) were incubated with I-ol (100 *μ*M) or CD (100 *μ*M) for 48 h. The SV Total RNA Isolation System® (Promega, USA) was used for cellular RNA extraction, where the extraction steps were carried out strictly according to the manufacturer's protocol. The extracted RNA was reverse transcribed into complementary DNA (cDNA) using random hexamer primers and M-MLV Reverse Transcriptase from Promega.

Reverse transcriptase-polymerase chain reactions were run in a LightCycler 480 instrument (Roche, Swiss). The QuantiTect SYBR Green PCR Kit (Qiagen, The Netherlands) was used for signal generation.

The primers were obtained from real-time primers (USA) as RNA arrays, named the “Human Cell Cycle Primer Library” and “Human Apoptosis Primer Library.” Hence, 176 validated primer pairs and primers against eight reference genes (ACTB, B2M, GAPDH, GUSB, HPRT1, PGK1, PPIA, and RPL13A) were analysed.

The fluorescence values after each cycle and the crossing point (Ct) were recorded using Roche's LightCycler 480 software and analysed using LinRegPCR (version 11) [21, 22]. The calculated mRNA-expression level of each target gene was compared to the arithmetic mean of the mRNA concentration of all eight reference genes. The ratio was calculated from the relative expression values in HepG2 cells treated with I-ol (100 *μ*M) or CD (100 *μ*M).

Due to the specific distribution of gene-expression values, a threshold was set for expression changes with Ct differences of larger than +5 and smaller than −7.5. The expression levels of the selected target genes were tested in three independent HepG2 cell samples, whereby three relative expression values were calculated for each target. A rank was assigned to each of these values within the respective biological sample, and a rank sum was calculated for each target. The higher the rank sum, the greater the repression induced by I-ol.

### 2.6. Regulation of Cell-Cycle Progression

The cell-cycle properties of HepG2 cells were analysed using the Click-iT® EdU Cell Cycle 405-Blue Flow Cytometry Assay Kit (Molecular Probes/Invitrogen, USA). Briefly, 2 × 10^6^ HepG2 cells were seeded into culture plates and incubated with medium (control group) or I-ol (the therapy group) at 3, 15, 30, 100, or 500 *μ*M I-ol for 48 h, or with 3, 100, or 500 *μ*M I-ol for 72 or 96 h. At the end of the incubation period, the cell concentrations of the different groups were adjusted to 1 × 10^6^ cells in 100 *μ*L PBS (1% BSA), and the experiments were performed in accordance with the manufacturer's instructions. Data generation and analysis were carried out with a FACSCanto II flow cytometer (BD Biosciences, USA).

After excluding cell debris and clusters of two or more cells, the fluorescence of 2 × 10^4^ single cells was depicted in a scatter chart, where the *y*-axis represented the 5-ethynyl-2′-deoxyuridine (EdU)-specific fluorescence on a logarithmic scale, and the *x*-axis represented the cell cycle-specific fluorescence on a linear scale.

### 2.7. Immunofluorescence Microscopy of Cultured HepG2 Cells

Four experimental groups were designed, and 5 × 10^4^ HepG2 cells were seeded into surface-coated eight-chamber cell culture object carriers (BD Biosciences, USA). These groups included (a) a control group incubated with Dulbecco's Modified Eagle's Medium, (b) a group incubated with I-ol (100 *μ*M), (c) a group incubated with CD (100 *μ*M), and (d) a negative-control group to assess the specificity of detection with the secondary antibody. After a 24-hour acclimation period, the corresponding experimental solutions were incubated for 48 h. Primary antibodies against the following proteins were used: Ki-67 (Abcam, United Kingdom, ab 833-500), cyclin D1 (Santa Cruz, USA, sc-20044), cleaved-caspase 3 (Cell Signaling Technology, USA, ASP 175), cMYC (Santa Cruz, USA, sc-764), and tumour necrosis factor (TNF)-alpha (Abcam, UK, ab 11560-50). As a secondary antibody, we used either a conjugate with Alexa Fluor 488 (green, Invitrogen, A 11034) or with Alexa Fluor 594 (red, Invitrogen, A11005) on the following day.

A fluorescein isothiocyanate-coupled primary antibody was used to detect TNF-alpha. Then, cells were incubated with 4′,6-diamino-2-phenylindole followed by mounting with ProLong Gold Antifade Reagent (Molecular Probes/Invitrogen, USA). Photographs were taken and compared qualitatively using an Area Scan camera with a charge-coupled device (CCD) sensor (Fuji, Japan).

### 2.8. p27 Immunohistochemistry of Cultured HepG2 Cells

HepG2 cells (4 × 10^6^) were seeded into culture plates. The four test groups described above were incubated with the indicated experimental solutions for 48 h. Subsequently, cell pellets were generated and fixed in 4% formaldehyde before being stored in an automatic drainage system overnight. Next, the cassettes were poured out with hot, liquid paraffin, and 2-*μ*m-thick paraffin sections were prepared and placed on an object carrier. The sections were deparaffinized in xylol and incubated in a descending series of alcohol and double-distilled water. A primary antibody against p27 (Santa Cruz Biotechnology, USA, sc-71813) was used for the immunohistochemical staining.

### 2.9. p27 Immunoblotting with Cultured HepG2 Cells

HepG2 cells (4 × 10^6^) were divided into four treatment groups as described above. The cell pellets were further processed using standard procedures to prepare them for sodium dodecyl sulphate-polyacrylamide gel electrophoresis and immunoblot analysis. Immunoblotting was performed with primary antibodies against p27 or beta-actin (a loading control) and a corresponding secondary antibody (Pierce/Thermo Fisher Scientific, USA, 1858415). The intensities of the protein bands were visualized with a CCD camera after the membrane was coated with a luminol buffer and reagent (Thermo Fisher Scientific, Wilmington, NC, USA) on a glass plate.

### 2.10. Haematoxylin/Eosin (H&E) Staining

H&E staining was performed with paraffin-embedded sections of CAM, explanted tumour tissues, as well as heart, liver, spleen, and kidney samples.

After the sections were deparaffinized, they were stained with haematoxylin. After incubation with eosin and xylene, the samples were visualized under a microscope.

### 2.11. Immunohistochemistry of CAM and Tumour Tissues

The deparaffinized sections from the CAM and animal studies were used for expression analysis with primary antibodies against the following proteins: desmin (Abcam, UK, 8470), cytokeratin (Agilent, USA, AE1/AE3), von Willebrand factor (vWF) (BioCore Medical Technologies, USA, N/A), Ki-67 (Abcam, UK, 833), and p27 (Santa Cruz Biotechnology, USA, 71813). Incubation with appropriate biotinylated secondary antibodies was followed by incubations with streptavidin-conjugated alkaline phosphatase Fast Red substrates. Counterstaining was performed with haematoxylin.

### 2.12. Terminal Deoxynucleotidyl Transferase (TdT) dUTP Nick-End Labelling (TUNEL) Assay of CAM and Tumour Tissues

Roche's In Situ Cell Death Detection Kit (Sigma Aldrich, USA, 11684795910) was used to perform TUNEL assays. Deparaffinized sections from the CAM and animal studies were processed according to the manufacturer's instructions. Counterstaining was performed with haematoxylin.

### 2.13. Statistical Analysis

We used the SPSS software tool (IBM SPSS Statistics, Version 22, USA) for statistical analysis and GraphPad Prism (Version 5, GraphPad Software, Inc., USA) to create the graphs.

The results of the cell culture and CAM experiments are shown as the mean ± SD. The results of the animal study are depicted as the individual values of the dependent variable (i.e., the measured pathological parameters), grouping around the mean value depicted with a horizontal line.

Statistical evaluation of the viability, cytotoxicity, and caspase-activity measurements, as well as of the data from the animal study, was carried out using two-way mixed analysis of variance (ANOVA), followed by a Bonferroni post hoc test. The data from the CAM and cell-cycle phase experiments were statistically analysed by one-way ANOVA and a subsequent simple-contrast procedure, followed by a Bonferroni post hoc test.

Time-series experiments were performed to study viability, cytotoxicity, and caspase activities in cultured HepG2 and DLD-1 cells, as well as in both animal studies. For each experimental group, we determined whether a statistically significant overall difference occurred in the measured parameters as a function of the incubation time, which was referred to as the “simple main effect of time.” This enabled differences in the measured parameters (e.g., cell viability) to be investigated following different incubation times without taking the individual drug concentrations into account.

Due to a clear assumption regarding the biological effect of the test compound I-ol, the hypotheses were considered as unilateral. In all cases, the significance levels were ^*∗*^*p* < 0.05 (significant), ^*∗∗*^*p* < 0.01 (highly significant), or ^*∗∗∗*^*p* < 0.001 (very highly significant).

Dose-dependent effects of I-ol were calculated by determining Pearson's correlation coefficient (*r*) and performing linear-regression analysis (adjusted *R*^2^) with the following statistical parameters: ^+^*p* < 0.05 (significant), ^++^*p* < 0.01 (highly significant), and ^+++^*p* < 0.001 (very highly significant).

To assess the usefulness of performing a linear adjustment of the data, 10 further nonlinear trend models were calculated using the SPSS “curve adjustment” function. During this validation step, no logically justifiable nonlinear alternatives emerged in a pharmacological sense. Therefore, nonlinear analyses were dispensed with and the parameters examined assumed to follow linear dose-response relationships.

## 3. Results

### 3.1. Viability, Cytotoxicity, and Caspase-Activity Assays in HepG2 and DLD-1 Cells

**Figure 1:**
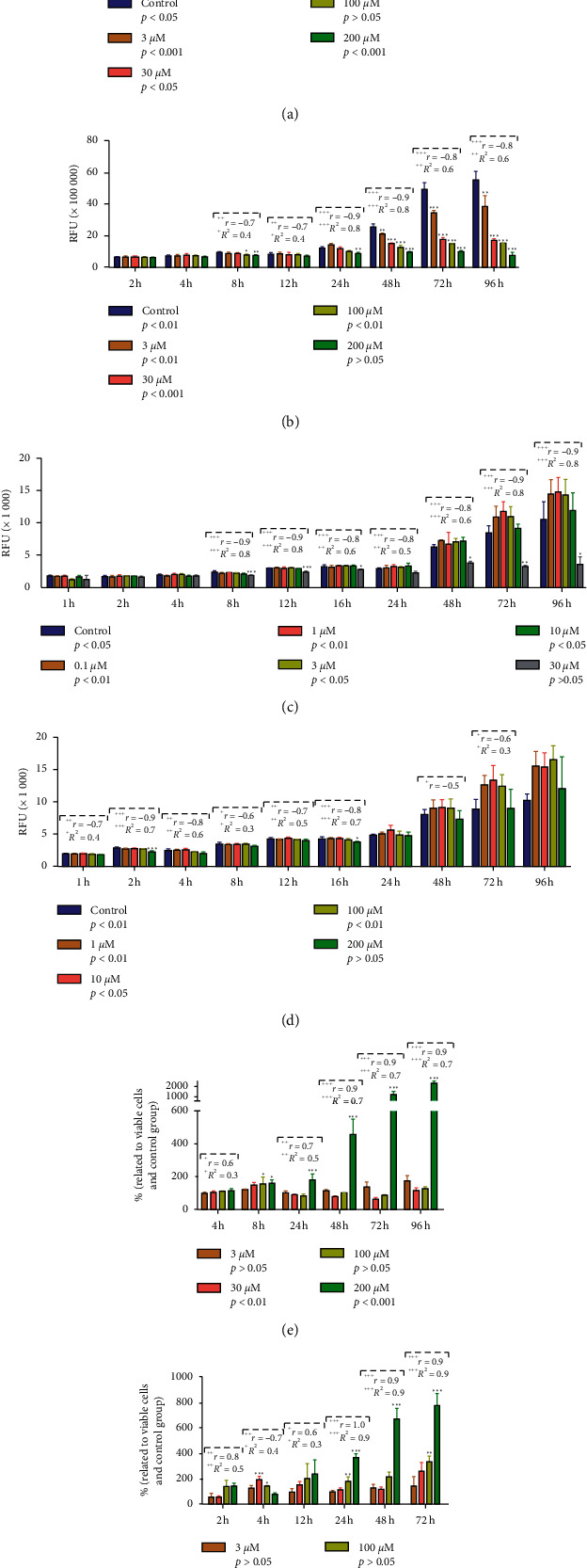
Viability, cytotoxicity, and caspase-activity assays in HepG2 and DLD-1 cells. (a) A very pronounced limitation of HepG2 viability in terms of a cytostatic effect already was observed after a 2-hour incubation with I-ol, as indicated by the relative fluorescence units (RFUs) observed. (b) Comparable effects on the viability of DLD-1 cells after 8 h with I-ol. (c) Reduced viabilities of HepG2 cells treated with smaller I-ol concentrations after 8 h only at the highest concentration. (d) No cytostatic effect on HepG2 cell viability was caused by treatment with cyclodextrin. (e) A strong increase in relative caspase activities of HepG2 cells treated with the highest I-ol concentration was found after an 8-hour incubation. (f) Maximum relative cytotoxic effects observed in HepG2 cells treated with the highest I-ol concentration after 24-hour incubation. The significance values from the “simple main effect of time” are shown below the group designations. The results are expressed as the mean ± SD. Statistical calculations were performed based on two-way mixed ANOVA, followed by Bonferroni's post hoc test. In all cases, the significance level was ^*∗*^*p* < 0.05 (significant), ^*∗∗*^*p* < 0.01 (highly significant), and ^*∗∗∗*^*p* < 0.001 (very highly significant). Dose-dependent effects of I-ol were calculated by determining Pearson's correlation coefficient (*r*) and performing linear-regression analysis (adjusted *R*^2^) with the following statistical parameters: ^+^*p* < 0.05 (significant), ^++^*p* < 0.01 (highly significant), and ^+++^*p* < 0.001 (very highly significant).

In all experiments, I-ol showed a strong concentration-dependent antiproliferative effect between concentrations ranging from 3 *μ*M to 200 *μ*M. This effect occurred in HepG2 cells (Figure 1(a)) after only a 2-hour incubation, in a very highly significant manner. The initial fluorescence values of HepG2 cells (measured after 2 h) treated with 100 *μ*M I-ol did not increase over the entire experimental period. In contrast, the fluorescence values of cells treated with 200 *μ*M showed a very highly significant reduction. In DLD-1 cells, a comparable effect occurred at an 8-hour incubation (Figure 1(b)) with 200 *μ*M I-ol showing a constant baseline.

In a further experiment, lower concentrations of I-ol (Figure 1(c)) were investigated in a range between 0.1 *μ*M and 30 *μ*M. In this case, only the highest concentration consistently showed an antiproliferative effect, with a constant baseline. However, this effect occurred at varying degrees of significance. Unlike the previous experiment, an I-ol concentration of 3 *μ*M showed no significant difference at any time point.

To study the influence of the carrier substance CD on viability, a concentration range of 1 *μ*M to 200 *μ*M was tested with HepG2 cells (Figure 1(d)). None of the concentrations showed an antiproliferative effect at any time point. Interestingly, 200 *μ*M CD showed no statistical significance over time. This might indicate that CD exerts an incipient antitumour effect in a dosage range outside of that tested in the current study.

Subsequently, we clarified whether I-ol has not only a cytostatic effect on tumour cells but also a cytotoxic or apoptotic effect. The measured values showed very highly significant effects after 24 h (Figure S6(b)) and 48 h (Figure S6(a)) but only at the highest concentration (200 *μ*M). These values are normalised to the number of living cells, with standardisation against the control group, enabling better assessment of the linear dose-dependent apoptotic (Figure 1(e)) and cytostatic (Figure 1(f)) effects. Furthermore, for both parameters, the highest I-ol dosage showed a significant (Figure 1(f), *p* < 0.01) or highly significant (Figure 1(e), *p* < 0.001) time-dependent effect.

In summary, I-ol showed a strong cytostatic effect on both cell lines, which could be described very well by a linear dose-response relationship. This observation was accompanied by cytotoxic and apoptotic effects at the high concentration ranges and with long incubation times. In contrast, CD did not show comparable effects.

### 3.2. mRNA-Expression Levels of Genes Related to Apoptosis and Cell-Cycle Progression in HepG2 Cells

**Figure 2:**
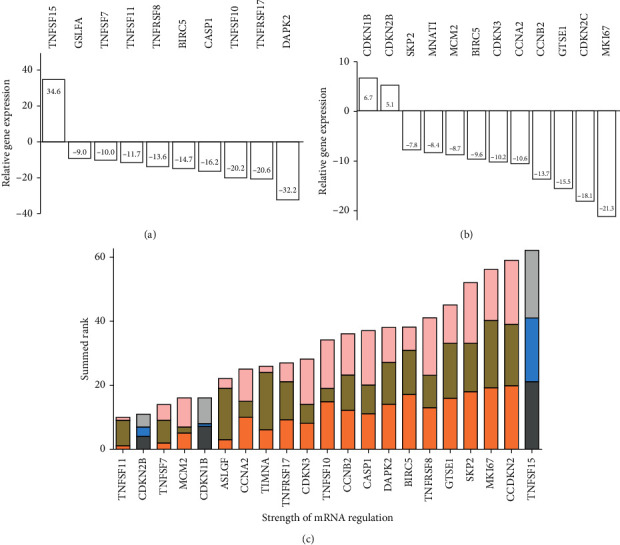
mRNA-expression levels of genes related to apoptosis and cell-cycle progression in HepG2 cells. (a, b) Cutoff values of ≥+5 and ≤−7.5 were determined, which resulted in the selection of 21 out of 88 analysed genes. These most dysregulated genes associated with cell-cycle progression and apoptosis were selected for further validation. BIRC5 expression was included in both arrays. Genes whose relative mRNA-expression levels could not be evaluated (due to no detectable amplification) were excluded from the analysis. (c) The relative mRNA-expression levels of the selected 21 genes were analysed in three independent experiments performed with HepG2 cells treated with I-ol (100 *μ*M) for 48 h. The expression level of each gene was ranked, where the highest expression levels corresponded with the highest rank. Finally, the sums of all three ranking values were calculated for each gene and are depicted as stacked columns. In concrete terms, this ranking indicated that TNFSF11 was the least expressed gene and that TNFSF15 the most expressed gene. Three differentially expressed genes (TNFSF15, CDKN1B, and CDKN2B) showed repressed mRNA expression compared to the control group treated with 100 *μ*M CD.

Initially, we determined the relative mRNA-expression levels of 88 genes that are considered essential for cell-cycle progression or apoptosis (Figure S7). This analysis was performed by screening a single biological sample of HepG2 cells. Overall, our results showed that the expression levels of more genes decreased after I-ol treatment than increased. The 21 most dysregulated genes associated with apoptosis (Figures 2(a) and 2(c)) and cell-cycle progression (Figures 2(b) and 2(d)) were selected for further analysis, using cutoffs of 5-fold induction or 7.5-fold repression. Three independent experiments were performed to assess how much the selected genes were dysregulated at the mRNA level (Figure 2(c)).

After completing the test series, the biological function of the TNFSF15 was poorly characterized concerning its function in the liver. We further analyse CDKN2B/p27 expression at the protein level, due to its central importance in regulating cell-cycle progression. We also studied MKI67/Ki-67 expression because it was the most well-known proliferation marker among the set of dysregulated genes.

### 3.3. Regulation of Cell-Cycle Progression

**Figure 3:**
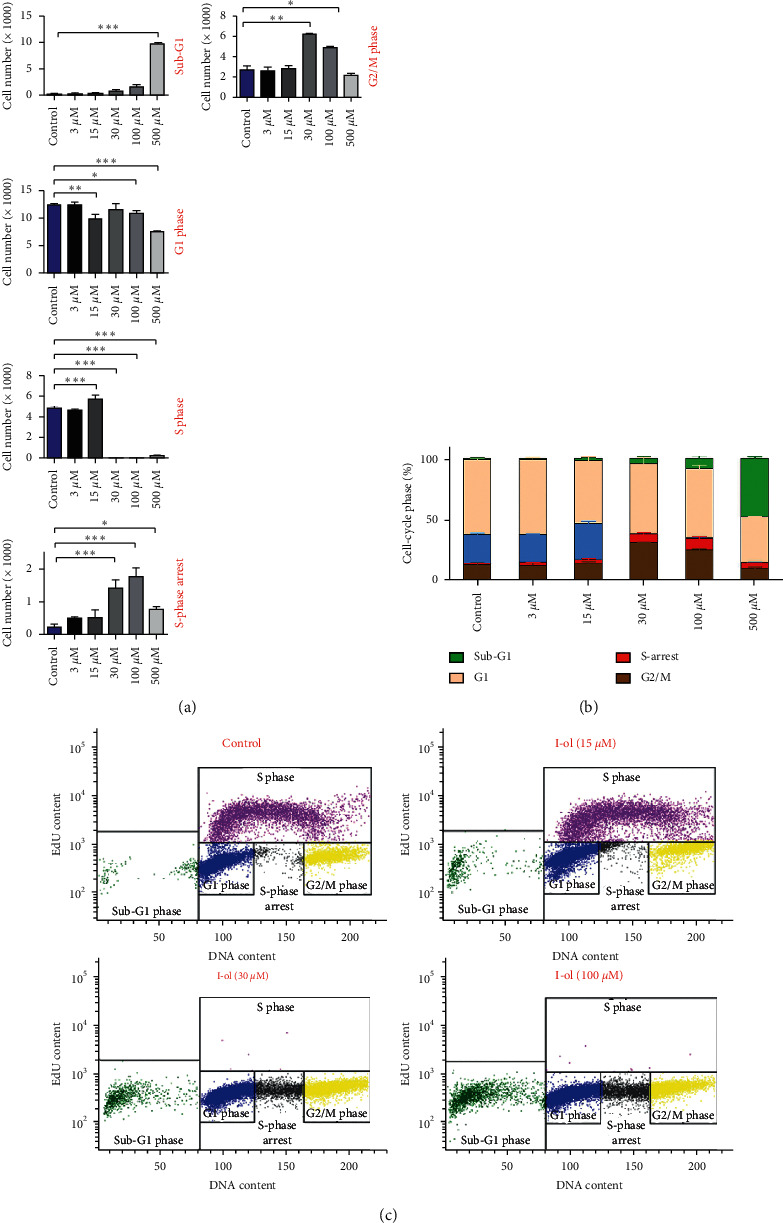
Regulation of cell-cycle progression. (a) Representation of each cell-cycle phase with increasing I-ol concentrations. A phase shift was observed at an I-ol concentration of 30 *μ*M. Specifically, we observed disappearance of cells in the S phase, a significant increase of S-phase arrest, and a large increase in the percentage of cells in G2/M phase and sub-G1 phase at the highest concentration. (b) Summary of the percentages of individual cells in each phase. (c) Representative scatter chart, where the *y*-axis represents the EdU-specific fluorescence on a logarithmic scale and the *x*-axis represents the cell cycle-specific fluorescence on a linear scale. The results after 48-hour incubation with I-ol are shown. After 72 or 96 h comparable results were obtained, those data are not shown to avoid redundancy. The results are shown as the mean ± SD. Statistical calculations were performed based on two-way mixed ANOVA, followed by Bonferroni's post hoc test. In all cases, the significance levels were ^*∗*^*p* < 0.05 (significant), ^*∗∗*^*p* < 0.01 (highly significant), or ^*∗∗∗*^*p* < 0.001 (very highly significant).

Due to the evaluated cytostatic effect of I-ol and its influence on the mRNA-expression levels of genes that regulate cell-cycle progression, we analysed the percentage of cells in each phase (Figure 3). Above an I-ol concentration of 30 *μ*M, there was almost no single cell actively engaged in DNA synthesis in the S phase. Cells that had already entered the S phase were arrested in their DNA synthesis capacity and virtually switched to a quiescent mode (S-phase arrest). Furthermore, an I-ol concentration of 500 *μ*M led to a massive increase in the number of apoptotic cells (sub-G1 phase). This finding explained the decrease in the absolute number of cells in the other cell-cycle phases (G1 phase and G2/M phase). All these effects were considered very highly significant, as determined by performing statistical analysis.

Despite the phase-specific effects of I-ol, none of the concentrations used showed a specific arrest at any phase in the cell cycle, which would have otherwise shown a large portion of the treated cells in one phase. This result remained constant even after incubation periods of 72 and 96 h (not shown).

### 3.4. Expression of Cellular Ki-67 and p27 in Cultured HepG2 Cells

Tumour cell arrest in the S phase should be accompanied by a decrease in protein expression of p27 and a corresponding increase of Ki-67, analogous to the observed mRNA-expression profiles.

**Figure 4:**
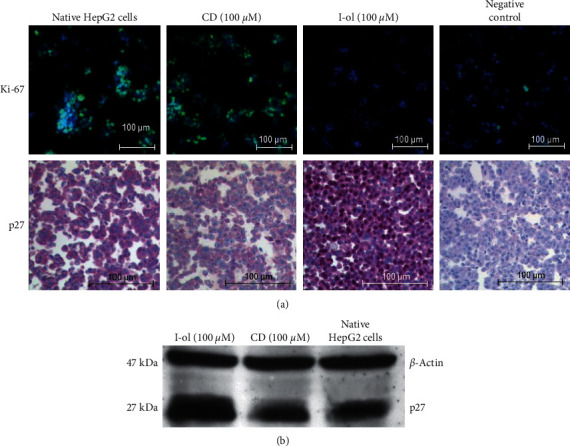
Expression of cellular Ki-67 and p27 in cultured HepG2 cells. (a) Immunofluorescent staining of Ki-67 and immunohistochemical staining of p27 in HepG2 cells. The magnification of the objective lens was 20-fold and that of the ocular lens was 10-fold, so the total magnification was 200-fold. Ki-67 protein expression was almost undetectable in cells treated with 100 *μ*M I-ol. In contrast, p27 expression increased by many times compared to both control groups. (b) Western blot analysis of p27 expression in protein extracts. The marked increase in p27 protein expression caused by I-ol treatment correlated very well with the previous results obtained with cultured cells. Representative images are shown.

The expression of Ki-67, cyclin D1, cleaved-caspase 3, cMYC, and TNF-alpha was studied via immunofluorescence microscopy with cultured HepG2 cells. Only Ki-67 showed a positive effect. In cell culture, CD (100 *μ*M) showed no significant effect on protein expression, whereas I-ol (100 *μ*M) treatment significantly decreased nuclear Ki-67 expression and led to halted proliferation. In terms of p27, we observed not only a sharp increase in total expression but also an increase in p27-positive nuclei compared to the control groups (Figure 4(a)). This observation was confirmed with protein extracts (Figure 4(b)), where the densitometric values showed an average of six-fold higher p27 levels in the treated group when compared to the two control groups. These results were consistent for all three biologically different samples tested.

### 3.5. HepG2 and DLD-1 Cell Growth in CAM Tissues

Qualitative and optical evaluations of H&E-stained cells showed that I-ol treatment strongly impacts tumour invasion into CAM tissues when compared to untreated cells. In the control groups, the tumour cells grew deep into the CAMs or even penetrated them, whereas the treated tumour cells remained on the surface (or appeared partially detached) and were characterized by distinct necrotic areas (Figure S8).

**Figure 5:**
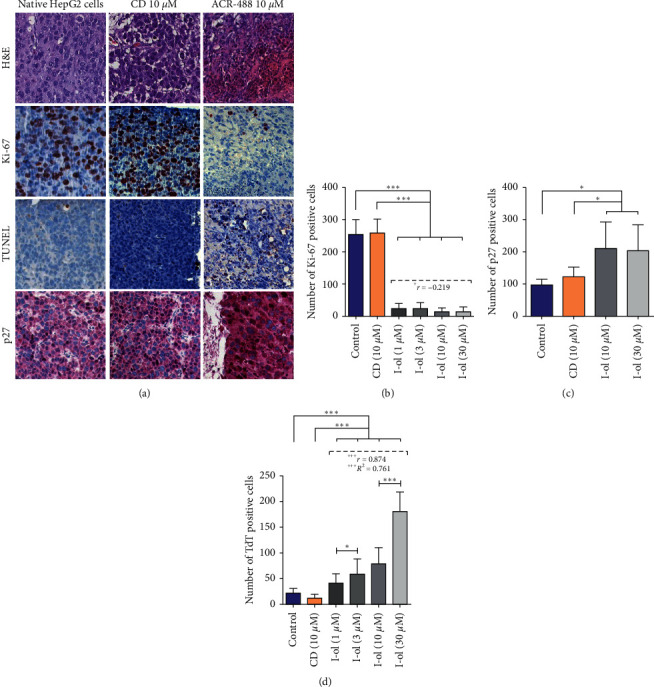
HepG2 cell growth in CAM tissues. (a) Representative images of H&E staining, Ki-67 and p27 immunohistochemistry, and TUNEL assay results. The magnification of the objective lens was 20-fold, and that of the ocular lens was 10-fold, such that the total magnification was 200-fold. (b) A massive reduction in the number of Ki-67-positive cells was observed after incubation with I-ol at the lowest concentration. (c) Induction of p27 protein expression. (d) Linear increase of apoptotic cells, compared with the control groups. The results are shown as the mean ± SD. Statistical calculations were performed based on two-way mixed ANOVA, followed by Bonferroni's post hoc test. In all cases, the significance level was ^*∗*^*p* < 0.05 (significant), ^*∗∗*^*p* < 0.01 (highly significant), or ^*∗∗*^*p* < 0.001 (very highly significant). Dose-dependent effects of I-ol were calculated by determining Pearson's correlation coefficient (*r*) and performing linear-regression analysis (adjusted *R*^2^), with the following statistical parameters: ^+^*p* < 0.05 (significant), ^++^*p* < 0.01 (highly significant), and ^+++^*p* < 0.001 (very highly significant).

**Figure 6:**
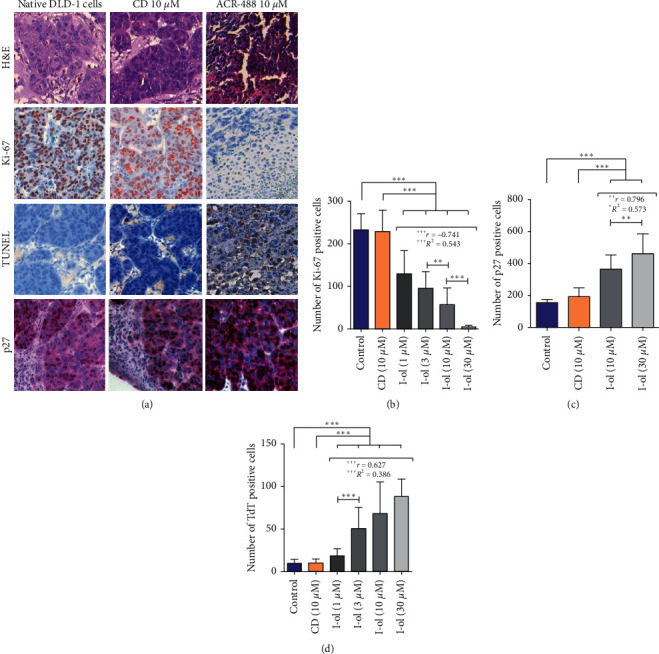
DLD-1 cell growth into CAM tissues. (a) Representative images of H&E staining, Ki-67 and p27 immunohistochemistry, and TUNEL assay results. The magnification of the objective lens was 20-fold, and that of the ocular lens was 10-fold, such that the total magnification was 200-fold. (b) A gradual reduction of Ki-67-positive cells was observed with increasing I-ol concentrations. (c) Induction of p27 protein expression. (d) Linear increase in the number of apoptotic cells, compared with the control groups. The results are shown as the mean ± SD. Statistical calculations were performed based on two-way mixed ANOVA, followed by Bonferroni's post hoc test. In all cases, the significance level was ^*∗*^*p* < 0.05 (significant), ^*∗∗*^*p* < 0.01 (highly significant), or ^*∗∗∗*^*p* < 0.001 (very highly significant). Dose-dependent effects of I-ol were calculated by determining Pearson's correlation coefficient (*r*) and performing linear-regression analysis (adjusted *R*^2^), with the following statistical parameters: ^+^*p* < 0.05 (significant), ^++^*p* < 0.01 (highly significant), and ^+++^*p* < 0.001 (very highly significant).

To study tumour growth in CAM tissues, p27 protein expression in tumour cells, and tumour cell apoptosis was examined by performing immunohistological staining (Figures 5(b) and 6(b)). The lowest concentration of 1 *μ*M caused a very highly significant reduction in the number of proliferating cells. The effect of I-ol was much more pronounced in HepG2 cells than in DLD-1 cells, but the latter cell type showed a strong linear dose-response relationship. At the highest dosage, almost no proliferating cells were detectable. Even the lowest concentration of I-ol significantly increased total p27 expression in HepG2 cells (Figure 5(c)) and very highly significantly increased p27 expression in DLD-1 cells (Figure 6(c)). These effects showed a pronounced linear dose-response relationship, compared with the control values. All I-ol concentrations showed a marked and very highly significant increase in the number of apoptotic cells with both cell lines. However, this effect was more pronounced in DLD-1 cells (Figures 5(d) and 6(d)). In both experiments, I-ol showed a strong linear dose-response relationship, which was very highly significant. In contrast, CD had no statistically significant effects, when compared to the control group.

### 3.6. Neovascularisation of CAM Tissues

To further evaluate the antitumour effect of I-ol, its effect on the vascular system was examined. The following tissue structures were studied immunohistochemically, which are part of the vascular system: desmin, cytokeratin, and vWF.

Visual inspection of the protein-expression levels in these structures did not reveal any clear influence of I-ol (Figure S9), so quantitative analysis was not performed.

### 3.7. Xenograft Animal Model

During both animal studies, six mice died prematurely (Figure S5). These mice were excluded from statistical analysis. The first animal study involved the death of two mice from the CD-control group and the death of three mice from the I-ol (3 mg/kg bw)-treated group. These deaths were strongly associated with anaesthesia and intravenous injection.

In the second animal study, the anaesthetic and injection technique were improved, which led to a decrease in the number of associated deaths. Thus, only one mouse in the CD-control group died.

The mice of the CD group bore clearly visible tumours on the last day of both animal studies. These tumours felt rough and were barely mobilizable under the skin. This suggested that the tumours infiltrated neighbouring structures such as the muscles of the shoulder girdle or the thoracic wall, which was confirmed by performing autopsies. The tumours of the treated mice were macroscopically smaller and could be readily moved in the subcutaneous adipose tissue. During the autopsies, infiltrative growth could only be detected in one animal, which was treated with I-ol (1 mg/kg bw).

The histological findings of the tumour preparations in both animal studies showed no significant differences between the groups in terms of H&E staining, immunohistochemical staining against Ki-67 and p27, and the TUNEL assay results (data not shown). These observations contrasted with a significant reduction in tumour masses and their macroscopic pathology in the treated groups.

**Figure 7:**
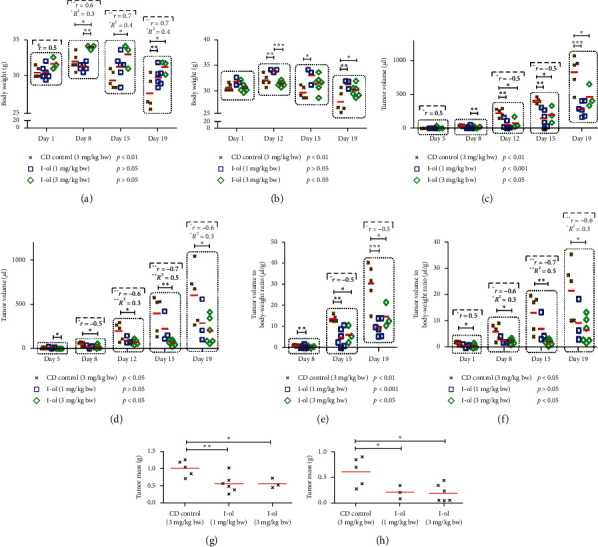
HepG2 cell-transplanted mice in a xenograft model. (a) Body-weight dynamics from animal study one. (b) Body-weight dynamics from animal study two. Increased body weights of the treated mice, compared to the control groups of both studies. (c) Tumour volumes from animal study one. (d) Tumour volumes from animal study two. Markedly reduced tumour volumes were observed in treated mice compared to the control groups, in both studies. (e) Tumour volume: bw ratios from animal study one. (f) Tumour volume: bw ratios from animal study two. The results are comparable to those described above. (g) Final tumour masses from animal study one. (h) Final tumour masses from animal study two. Over a two-fold decrease in the final tumour masses was observed in both studies. The significance values determined as the “simple main effect of time” are shown below the group designations. The results are depicted as individual values of the dependent variable (i.e., the measured pathological parameters) grouped around the mean value, which is depicted with a horizontal red line. Statistical calculations were performed based on two-way mixed ANOVA, followed by Bonferroni's post hoc test. In all cases, the significance levels were ^*∗*^*p* < 0.05 (significant), ^*∗∗*^*p* < 0.01 (highly significant), or ^*∗∗∗*^*p* < 0.001 (very highly significant). Dose-dependent effects of I-ol were calculated by determining Pearson's correlation coefficient (*r*) and performing linear-regression analysis (adjusted *R*^2^), with the following statistical parameters: ^+^*p* < 0.05 (significant), ^++^*p* < 0.01 (highly significant), and ^+++^*p* < 0.001 (very highly significant).

In both treatment groups, I-ol led to a significant increase in body weight (Figures 7(a) and 7(b)) compared to the CD-control group beginning on day 8 (animal study one) or day 12 (animal study two). The measured values did not differ statistically from each other over the entire experimental period, which suggested that the mice maintained a constant body weight. An exception was the group treated with 3 mg/kg bw I-ol in the second study, where the treatment time significantly influenced the body weight. Beginning on day 8 of treatment, both doses of I-ol markedly reduced the tumour volumes (Figures 7(c) and 7(d)), which indicated a linear dose-response relationship, particularly in the second study. In this case, the treatment duration had no statistically significant effect, which suggested that the tumour volumes were constant. Analysing relationships between tumour volumes and body weights (Figures 7(e) and 7(f)) revealed statistically significant differences. In both animal studies, I-ol caused at least a two-fold decrease in the final tumour mass (Figures 7(g) and 7(h)).

To assess any possible toxicological side effects of I-ol, we conducted histopathological examinations of representative organs, including the liver, kidneys, spleen, and heart. H&E staining of the tissue sections consistently revealed no evidence of pathology, based on morphology (Figure S10). This finding confirmed the results of our acute-toxicity study conducted in rats (Table S2).

## 4. Discussion

When this study was designed, the antitumour effect of I-ol was completely unknown. A model HCC cell line (HepG2 cells) and a model colorectal cancer cell line (DLD-1 cells) were used because the development and progression of both diseases are associated with CYP2E1 [16, 23]. Because we have already shown that I-ol was effective in treating ASH [24] and NASH (unpublished data), I-ol was tested for efficacy against hepatocarcinogenesis. A further rationale for this proof-of-concept study was the potential for evaluating the possible extension of I-ol for treating the lethal end stage of FLD. Due to the lack of clinical and laboratory algorithms, the diagnosis of HCC in the setting of FLD occurs much later than in hepatitis C virus (HCV)-associated tumorigenesis. This circumstance leads to a larger tumour burden and increased infiltrative growth in FLD patients, which explains their increased mortality [25].

This work revealed cytostatic effects of the CYP2E1-specific inhibitor I-ol in HepG2 and DLD-1 cells. Only the high concentrations showed cytotoxic effects, which are partly caused by apoptosis. In the mice of the therapy groups, I-ol also showed an anticachectic effect, as the body weight of the animals remained constant over the test period. Furthermore, the organs of these animals did not show any pathological changes. This result is consistent with the very low acute toxicity of I-ol.

The therapeutic effect could be explained by a general arrest of cell-cycle progression, which particularly affected the S phase. Increased p27 expression seemed to be a major driver of the described effects.

Extensive screening of mRNA-expression levels for genes associated with apoptosis and cell-cycle progression revealed increased transcription of only three genes after incubation of I-ol: CDKN1B (p27kip), CDKN2B (p15ink4B), and TNFSF15 (a TNF receptor ligand). p27 and p15 are inhibitors of cyclin-dependent kinases, and the manifold importance of which is increasingly being investigated [26, 27]. Among other things, the expression levels of these proteins were decreased in several types of tumours and neoplastic tissues, which was associated with a poor prognosis [28]. p27 and another family member (p16ink4A) are also frequently inactivated in HCC [29, 30]. High p27 expression has been associated with high survival rates. In contrast, reduced p27 expression is considered an independent marker of poor prognosis and often occurs during the advanced stage of HCC [31, 32]. It was also shown that the p27 and p15 expressions were regulated by the demethylase KDM5B (also known as JARID1B). Silencing KDM5B promoted both p15 and p27 expressions by increasing histone H3K4 trimethylation in their promoters [33], which simultaneously led to G1-/S-phase arrest [27]. KDM5B expression was upregulated in HCC cells and tissues but not in the periphery of the tumour tissue [34, 35]. Furthermore, high KDM5B expression was positively correlated with metastasis and decreased the overall survival of patients with HCC [36]. Pilot experiments revealed an antimigratory effect of I-ol at concentrations ranging from 0.1 to 10 *μ*M (Figure S11). Therefore, elevated p27 expression can be considered a “therapeutic” success upon treatment with I-ol as it exhibited an effect similar to that observed after knocking down KDM5B expression.

The lack of staining-intensity differences for vWF, cytokeratin, and desmin showed that a reduced vascular supply for the tumours through the chorioallantoic membrane was not the cause of cell death. The reduced invasiveness of tumour growth into CAM tissues by I-ol may be due to increased p27 expression and impairment of its cytosolic function [37].

However, there is currently no evidence supporting the relationship between CYP2E1 activity and p27 expression, in particular its importance in cell-cycle regulation and its role in ROS generation. A recent publication showed that ROS generated by CYP2E1 degraded polyunsaturated fatty acids to 4-hydroxynonenal, which caused Akt dephosphorylation [38]. Consequently, it may be anticipated that the p27 and RB expression or activation of the associated signaling pathway was reduced. Indeed, it has been shown that ROS can induce dephosphorylation of the cell-cycle-related protein pRb and its family members (p107 and p130) due to the activity of protein phosphatase 2A, leading to reduced DNA synthesis [39].

One of our studies showed that ROS production in cultured HepG2 cells was low and was accompanied by low CYP2E1 enzymatic activity. Treatment with 40 *μ*M I-ol led to a reduction of both parameters, but these differences were not statistically significant [24]. This observation correlates with the finding that both parameters reach their maximum in cirrhotic livers but decrease to a minimum during the transition to HCC development [17, 18].

Current data suggest that CYP2E1 does not play a central role in liver tissues after they have undergone malignant transformation. Consequently, the antitumour effect of I-ol should not be induced by its enzymatic inhibition, but instead through a mechanism that drives p27 expression and leads to tumour cell cytostasis. The importance of CYP2E1 as a drug target could be central, particularly in cirrhotic livers since its enzymatic activity and ROS formation appears to be key drivers of hepatocarcinogenesis. Thus, I-ol can potentially be used to stabilize hepatocytes that have not yet been malignantly transformed, thereby preventing the transition from steatohepatitis or cirrhosis to HCC, as well as arresting tumour cells in their progression.

Another perspective arises from the indication that the oral multikinase inhibitors sorafenib and regorafenib used in first-line therapy lead to an increase of CYP2E1 protein expression. Whether this observation leads to a notable elevation in enzyme activity and ROS formation should be the subject of further research. Possibly, an add-on therapy with I-ol could suppress this reverse regulation in tumour tissue and lead to an improved therapy outcome [40].

Both approaches would represent a novelty in the extremely limited drug-therapy options for HCC.

## 5. Conclusions

The cytostatic effect of the new CYP2E1 inhibitor, I-ol, on hepatocellular carcinoma progression is mainly mediated by the cell-cycle regulator, p27. In the absence of off-target mediated organ damage, I-ol may represent an innovative therapeutic option in the treatment of advanced FLD.

## Data Availability

The data used to support the findings of this study may be released upon application to the corresponding author, who can be contacted via tdiesinger@gmx.de.

## Supplementary Materials

Supplementary Materials The supplementary material file includes supplementary figures and tables containing detailed information on various experiments that support the data in the manuscript.
