# Supplementary material for: A New CYP2E1 Inhibitor, 12-Imidazolyl-1-dodecanol, Represents a Potential Treatment for Hepatocellular Carcinoma
Source: Can J Gastroenterol Hepatol. 2021 Feb 2;2021:8854432. doi: 10.1155/2021/8854432 (PMC7872744; doi:10.1155/2021/8854432)
Supplement: Supplementary Materials — The supplementary material file includes supplementary figures and tables containing detailed information on various experiments that support the data in the manuscript. [file 8854432.f1.docx]

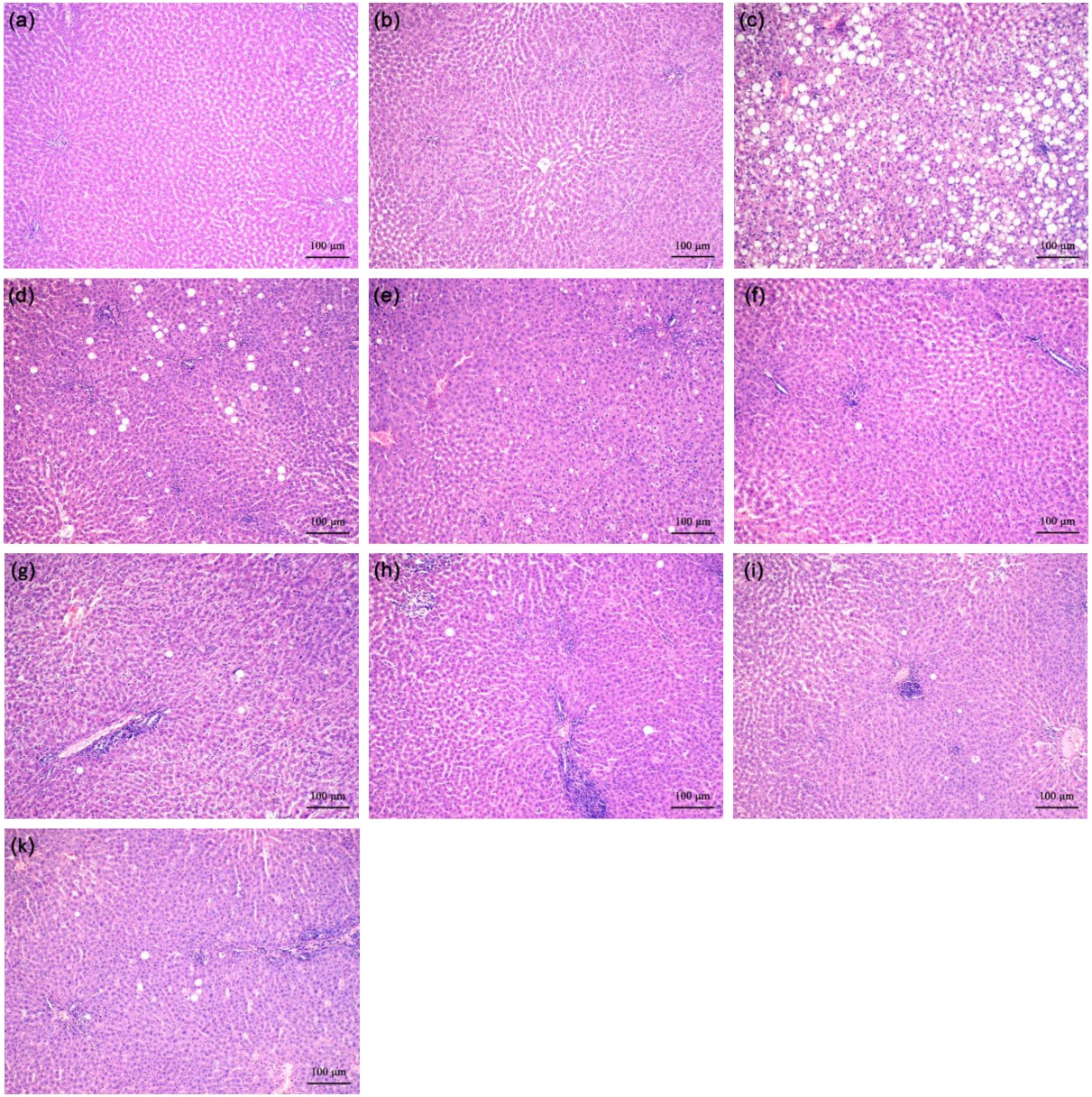
**Supplementary Material**

Figure S1: Representative histological images of liver sections generated in a proof-of-concept study of ASH performed with rats. The rats were divided into the following treatment groups: (a) Control group. (b) Isocaloric diet without alcohol. (c) Disease group with drinking water containing 5% alcohol (EtOH). (d) I-ol (0.4 mg/kg bw) + EtOH. (e) I-ol (4 mg/kg bw) + EtOH. (f) I-ol (40 mg/kg bw) + EtOH. (g) 1-imidazolyldodecane (I-an; 0.4 mg/kg bw) + EtOH. (h) I-an (4 mg/kg bw) + EtOH. (i) I-an (40 mg/kg bw) + EtOH. (k) Ursodeoxycholic acid (UDCA; 40 mg/kg bw) + EtOH. UDCA is a hepatoprotector that was used as reference drug. All sections were stained with HE. The magnification of the objective and ocular lenses was 10-fold, such that the total magnification was 100-fold.


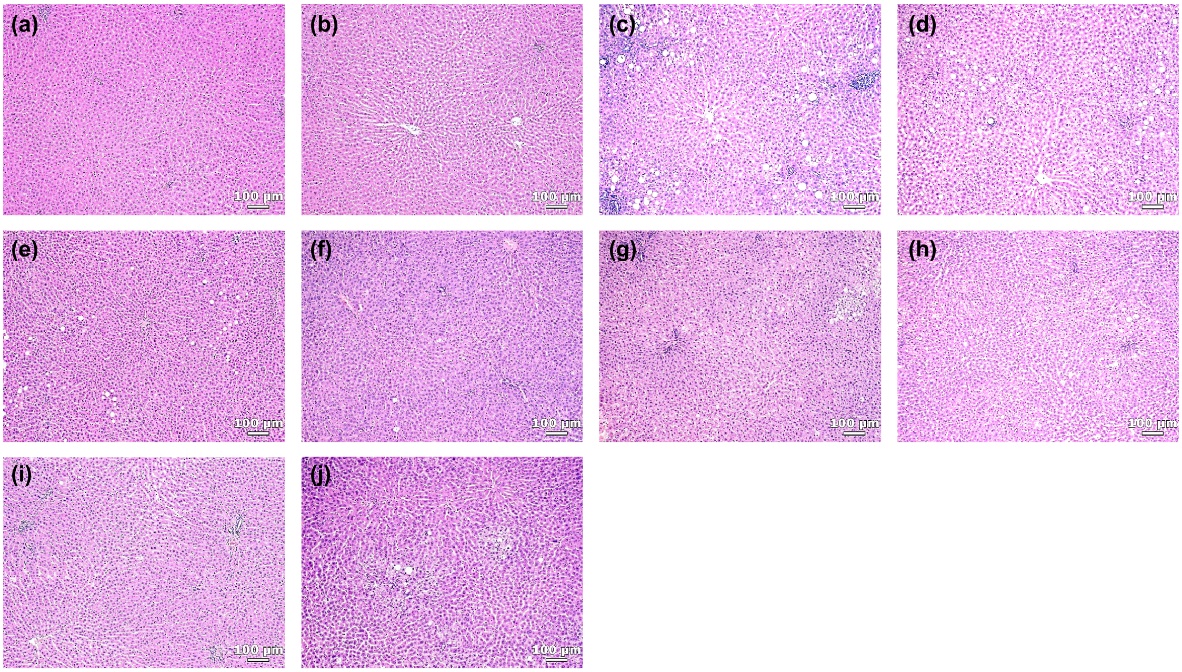
Figure S2: Representative histological images of liver sections generated in a proof-of-concept study of NASH performed with rats. The rats were divided into the following treatment groups: (a) Control group. (b) Liquid diet with a normal fat content. (c) Liquid diet with a high-fat content (HFD). (d) I-ol (0.4 mg/kg bw) + HFD. (e) I-ol (4 mg/kg bw) + HFD. (f) I-ol (40 mg/kg bw) + HFD. (g) UDCA (40 mg/kg bw) + HFD. (g) I-an (0.4 mg/kg bw) + HFD. (h) I-an (4 mg/kg bw) + HFD. (i) I-an (40 mg/kg bw) + HFD. (k) UDCA (40 mg/kg bw) + HFD. Nine sections of each liver were prepared from six or eight animals per group. The sections are stained with HE. The magnification of the objective and ocular lenses was 10-fold, such that the total magnification was 100-fold.


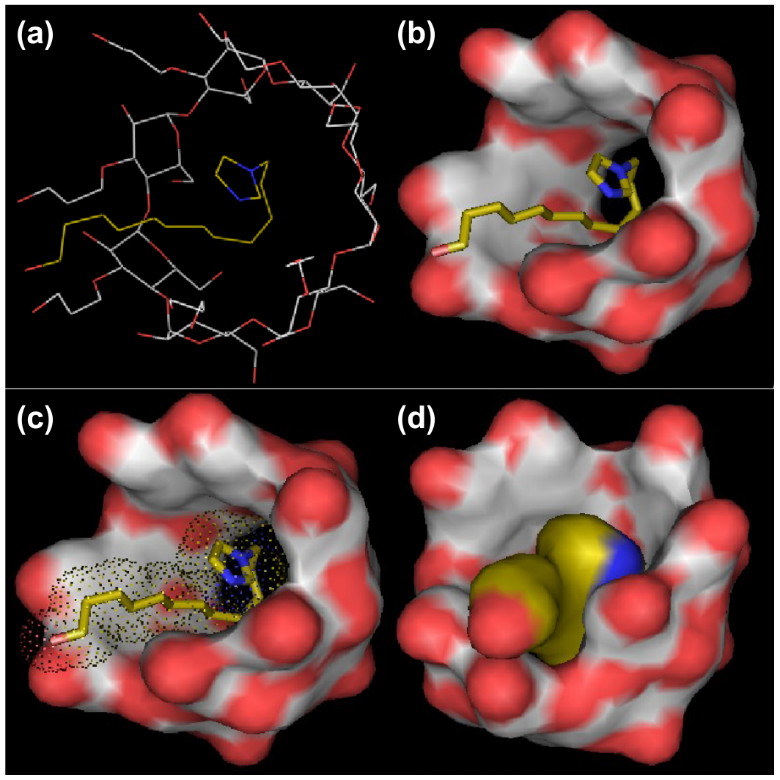
Figure S3: Schematic illustration of the interaction between I-ol und HPβCD. (a) Wire-frame mode for both molecules. (b) Stick mode for I-ol and surface mode for HPβCD. (c, d) Surface mode for both molecules, where the shading is defined as follows: carbon atoms of HPβCD (white), carbon atoms of I-ol (yellow), oxygen (red), and nitrogen (blue). The illustrated conformation represents one of the most thermodynamically stable conformations between both interacting partners. The interior of the structure encloses a hydrophobic cavity with a dimeter of 260 nm. The hydrophobic region of I-ol fits entirely into this cavity due to its flexible structure. The hydrophilic outer surface guarantees solubility in aqueous environments, such as buffer systems, cell culture medium, and blood. The hydroxyl group of I-ol generates polar interactions with hydroxyl groups on the outer surface of HPβCD (more precisely: a hydrogen bond, which is not shown here). This bridge confers additional stability to the complex once formed, which increases the solubility of I-ol.


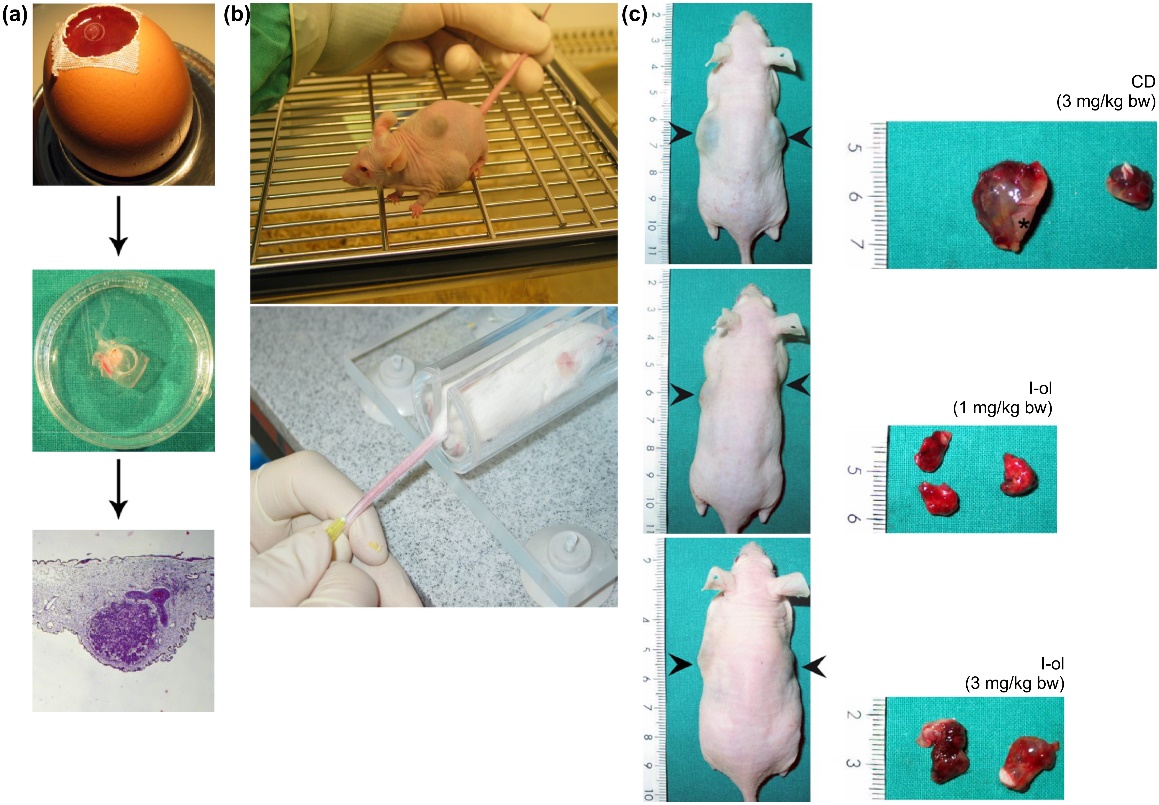
Figure S4: Handling of CAM tissues and xenograft mouse models. (a) Representative image sequence for the experimental process used with the CAM method. After opening the egg shell, a silicone ring was applied. The human tumour cells were placed within this ring. After a defined incubation phase, the tissue enclosed by the ring was removed and histologically examined. (b) Representative pictures of tumour-bearing nude mice and injection into the tail vein. (c) Representative images of a mouse from each experimental group, as well as their explanted tumours at the end of the animal study. The scales represent centimetres, the arrowheads point to the tumours in situ, and the star marks a region of the chest wall that was covered with tumour tissue.



Figure S5: Scheme of animal studies. For reasons related to professional handling, two animal experiments (AEs) were carried out consecutively. Thirty-four mice were used and divided between the three experimental groups at the beginning of the experiment. Six mice died during the studies, and those mice were not included in the evaluations.


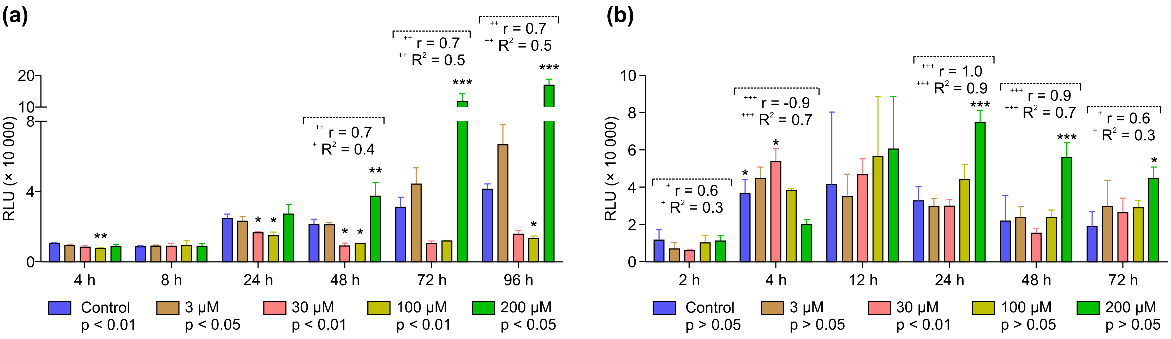
Figure S6: Absolute cytotoxicity and caspase activity of HepG2 cells. (a) Strongly increased caspase activities in HepG2 cells treated with the highest I-ol concentration were observed at a 48-h incubation. (b) Cytotoxic effects in HepG2 cells treated with I-ol. The results are expressed as the mean ± SD. Statistical calculations were performed based on two-way mixed ANOVA, followed by Bonferroni’s post-hoc test. In all cases, the significance level was *p < 0.05 (significant), **p < 0.01 (highly significant), or ***p < 0.001 (very highly significant). Dose-dependent effects of I-ol were calculated by determining Pearson’s correlation coefficient (r) and by performing linear-regression analysis (adjusted R^2^), with the following statistical parameters: +p < 0.05 (significant), ++p < 0.01 (highly significant), and +++p < 0.001 (very highly significant).


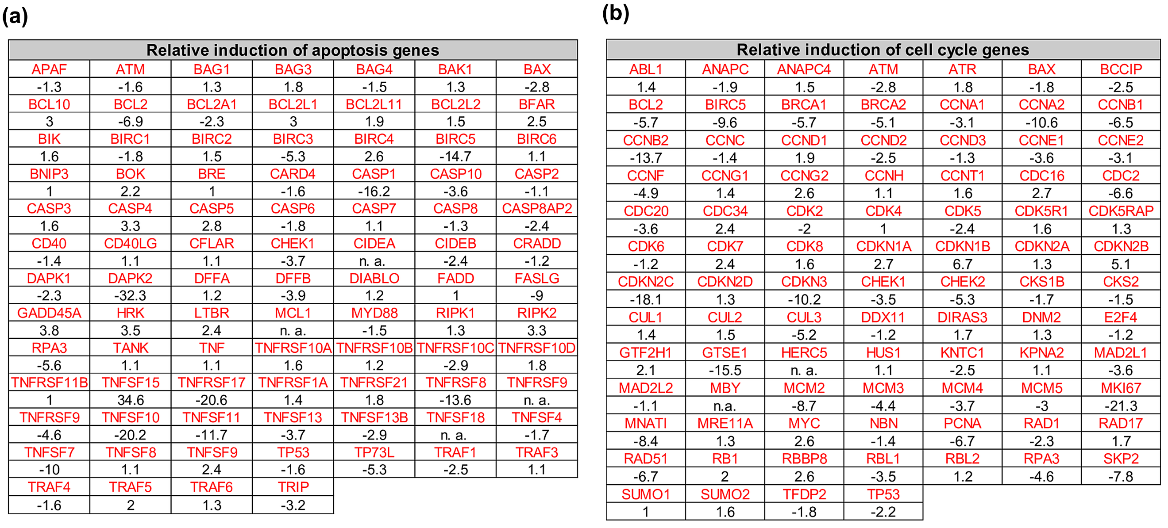
Figure S7: Relative induction of genes associated with apoptosis and cell-cycle progression. mRNA-expression levels of genes related to apoptosis (a) and cell-cycle progression (b) in HepG2 cells. The cell-cycle and apoptosis PCR arrays each consisted of primers for amplifying 88 pathway-specific and 8 reference genes. HepG2 cells were separately incubated with I-ol (100 µM) or CD (100 µM) for 48 h. The relative mRNA-expression levels were calculated using LinRegPCR software, where positive values reflected gene induction and negative values reflected gene repression.


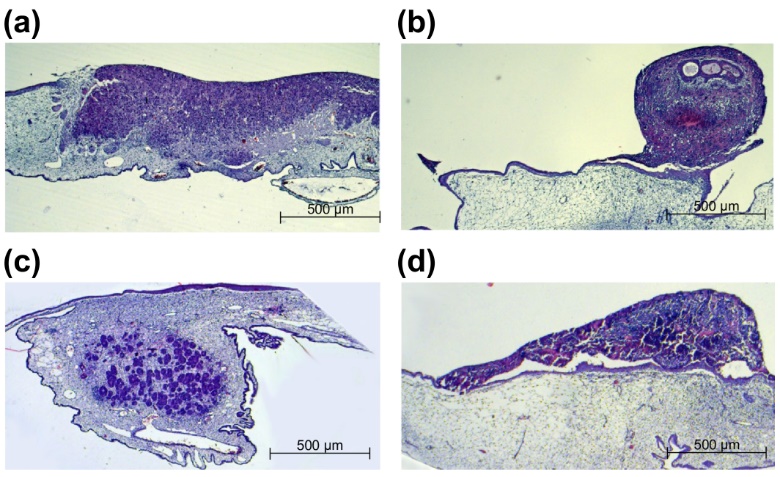
Figure S8: Representative histological images of CAM tissues after HE staining. (a) Untreated HepG2 cells. (b) HepG2 cells treated with I-ol (10 µM). (c) Untreated DLD-1 cells. (d) DLD-1 cells treated with I-ol (10 µM). Incubating both tumour cell lines with I-ol changed their growth behaviours within the CAM tissues, as characterized by significantly lower invasiveness, increased necrotic tissue, and shrinkage of the tumour mass. The magnification of the objective lens was 5-fold and that of the ocular lens was 10-fold, such that the total magnification was 50-fold.


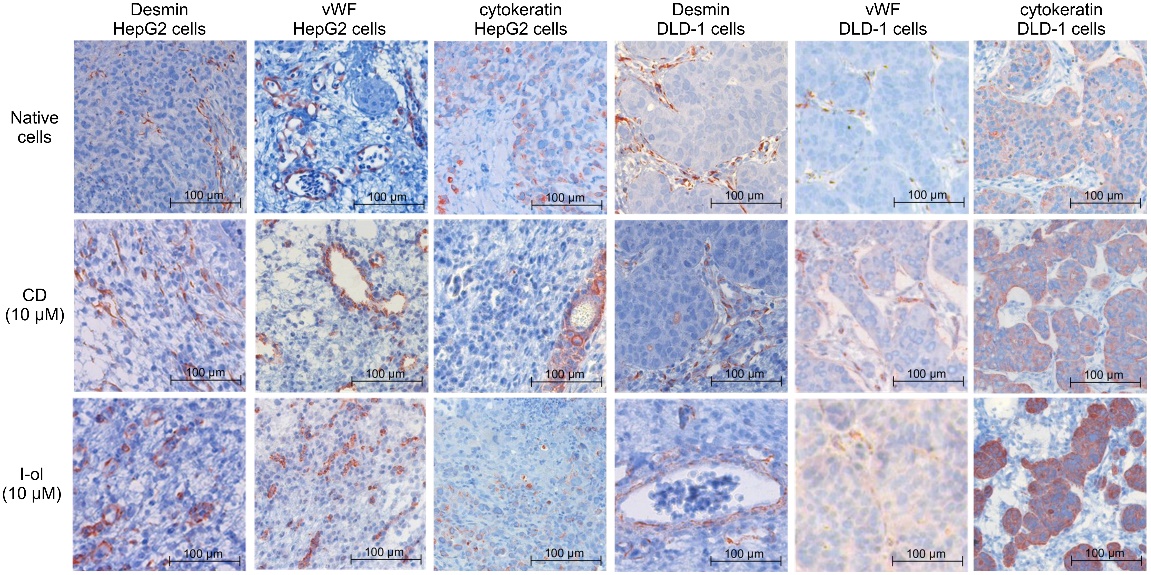
Figure S9: Neovascularization of CAM tissues. Representative images obtained after immunohistochemical staining for desmin, vWF, and cytokeratin. The magnification of the objective lens was 20-fold and that of the ocular lens was 10-fold, such that the total magnification was 200-fold. Whether the formation of new vessels in CAM tissues was induced by transplanting the HepG2 and DLD-1 cells should be investigated further. In particular, the question of whether incubation with I-ol could have prevented such neovascularization is of interest. Increased vascularization in the untreated tumour samples or inhibition by I-ol was not observed.


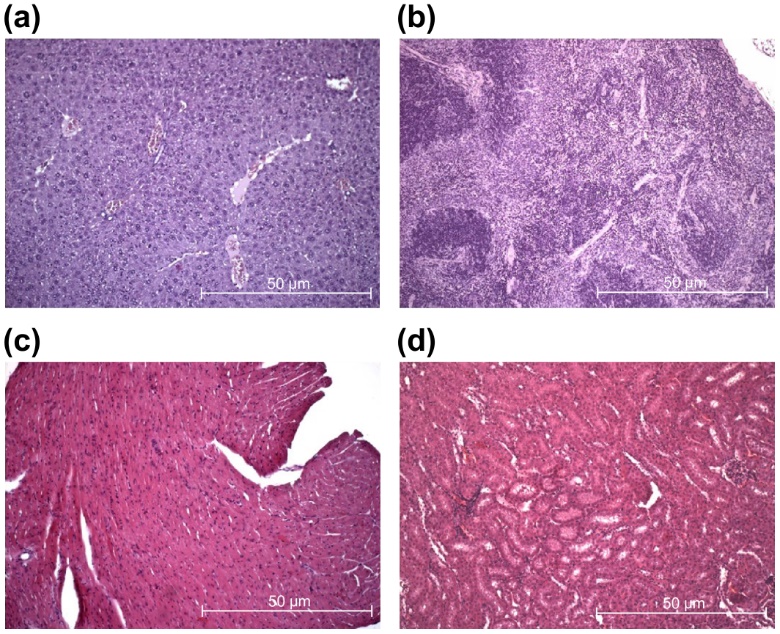
Figure S10: Representative histological images of internal organs. Sections were prepared from organs of mice treated with I-ol (3 mg/kg bw) and stained with HE. The magnification of the objective and ocular lenses was 10-fold, such. that the total magnification was 100-fold. (a) Liver (b) Spleen (c) Heart (d) Kidney. All sections were characterized by a physiologically typical tissue architecture.


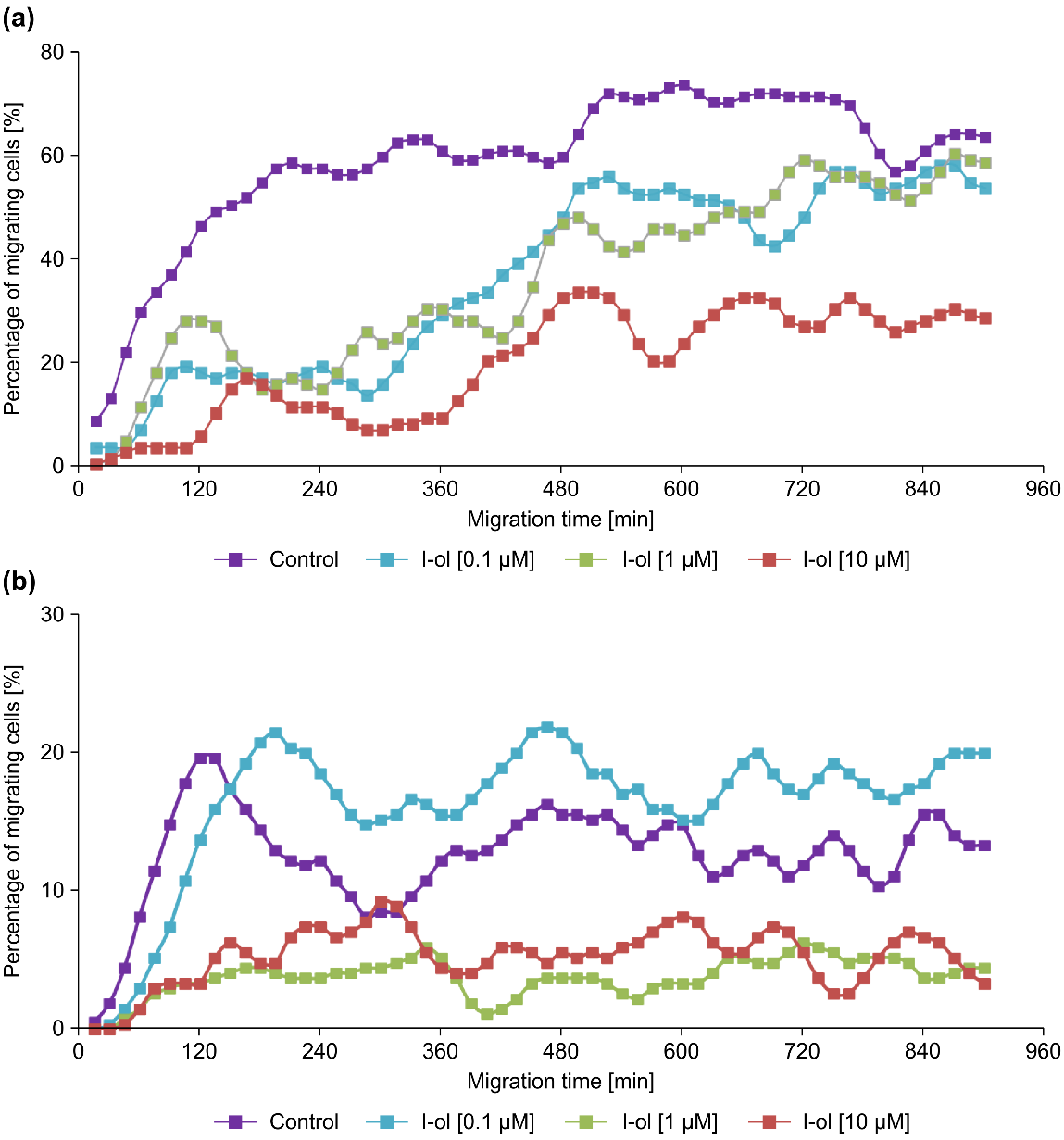
Figure S11: The effect of I-ol on the migratory activity of HepG2 cells. The anti-metastatic effect of I-ol was evaluated specifically in terms of the first step of the metastatic cascade (the ability of tumour cells to migrate). The experiment was carried out using time-lapse video microscopy. This approach facilitated a precise analysis of tumour cell movement in a three-dimensional collagen matrix using a cell-tracking software program developed in-house. The method used was described by Niggemann et al. [33]. The average percentage of migrating HepG2 cells was calculated for each group at 15-min intervals. (a) The migratory behaviour of the HepG2 cell line was analysed as a function of different I-ol concentrations. The concentrations of 0.1 µM and 1.0 µM cause a visible anti-migratory effect on the HepG2 cells, which did not obviously differ from each other. However, increasing the I-ol concentration to 10 µM led to a clearly distinguishable reduction of migration. (b) Incubation with pure cyclodextrin resulted in a pro-migratory effect. I-ol, dissolved in 0.003% dimethyl sulphoxide, shows a strong anti-migratory effect, which was slightly attenuated by complexation with CD.


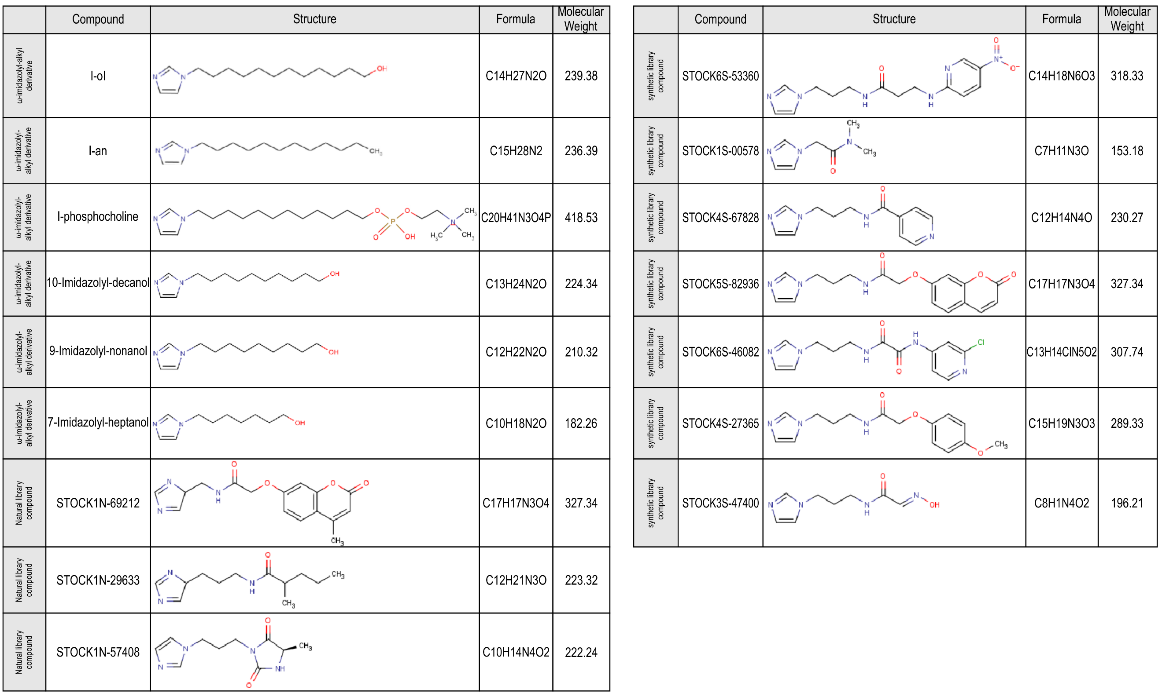
Table S1: All in vitro-tested compounds containing an imidazole ring system. The chemical compounds shown are CYP2E1 inhibitors that have been investigated in the context of rational drug development for treating ASH and NASH. I-an is 1-imidazolyldodecane, the dehydroxylated form of I-ol, which has also been tested as an active compound in animal models.



Table S2: Acute toxicity study for I-ol. Experimental design: Thirty-five male CD^®^/Crl:CD(SD) rats (Charles River Deutschland GmbH) were used after randomisation. Five rats were selected for each concentration. In addition, a control group was formed using five rats that received only the hydroxypropyl methylcellulose gel, in which I-ol was dissolved for a peroral application using a gastric tube. Each animal was observed for 14 days, so that deviating behaviour from the norm could be systematically recorded. Observations were made before the application; at 0, 5, 15, 30, and 60 min after the start of the application; and at 3, 6, and 24 h after the start of the application. These observations were followed by subsequent observations at least once/day over a period of 14 days. The observations were focused on changes in skin, fur, eyes, mucous membranes, respiration, circulation, the autonomous and central nervous systems, somatomotor activity, and behavioural patterns. Attention was also paid to possible tremors, convulsions, salivation, diarrhoea, lethargy, sleep, and coma. Mortality observations were made at least once/day to minimise the loss of animals during the study. At the end of the experiments, all surviving animals were sacrificed, dissected, and examined macroscopically. All gross pathological changes were recorded. Microscopic examination was performed for all organs showing obvious lesions obtained from animals that survived for 24 h or longer. Autopsy and macroscopic inspection of prematurely deceased animals were performed as soon as possible after death. Results: (a) I-ol treatment showed no acute toxicity up to a concentration of 1000 mg/kg bw. At this maximum dose, reduced motility was observed in parallel with reduced muscle tone, ataxia, and dyspnoea, whereas no inhibition of bw gain or necropsy findings were detected. None of the 30 rats died; therefore, the lowest lethal dose, as well as the LD_50_ dose (14 days), was by definition greater than 1000 mg/kg bw (b) Summary of the data generated in the acute-toxicity study.
